# Supplementary material for: General Practitioners’ Own Traumatic Experiences and Their Skills in Addressing Patients’ Past History of Adversities: A Cross-Sectional Study in Portugal
Source: Healthcare (Basel). 2023 Sep 1;11(17):2450. doi: 10.3390/healthcare11172450 (PMC10487406; doi:10.3390/healthcare11172450)
Supplement: Supplementary file 1 [file healthcare-11-02450-s001.zip › healthcare-2559066-supplementary.pdf]

Supplementary Material S1

Eight initial questions used in the fourth section of the survey

1. Do you routinely ask your patients about their personal history of exposure to traumatic events and adverse childhood experiences?

   [ ] Yes

   [ ] No

2. Do you ask your patients about their personal history of exposure to traumatic events and adverse childhood experiences only in some specific contexts?

   [ ] Yes

   [ ] No

3. If you answered "Yes" to the previous question, in which contexts do you do ask your patients? You can select more than one option.

   [ ] Depressive and/or anxious symptoms

   [ ] Chronic illness

   [ ] A delicate family context

   [ ] Situations of multimorbidity

   [ ] With very frequent attenders

   Other____

4. Who do you think should first broach this topic?

   [ ] The physician

   [ ] The patient

   [ ] It can be either one or the other, depending on the situation

5. How important do you think it is for your patients to address their past history of exposure to traumatic events and adverse childhood experiences?

[ ] Not important

[ ] Of little importance

[ ] Indifferent

[ ] Important

[ ] Very important

6. Do you think your patients would like you to routinely discuss their past history of traumatic events and adverse childhood experiences?

   [ ] Totally disagree

   [ ] Disagree

   [ ] Neither agree or disagree

   [ ] Agree

   [ ] Totally agree

7. What reasons can you identify for physicians not addressing these issues routinely?

   [ ] Shame

   [ ] Refusal

   [ ] A lack of consultation time

   [ ] Not important for the consultation

   [ ] Fear of causing more harm or suffering for the patient

   [ ] Fear of infringing the doctor-patient relationship

   [ ] Difficulty in providing an empathetic and sufficiently strong response to the strong emotions that surface when patients share these issues

   [ ] A lack of training in clinical communication

   [ ] A feeling that you haven't received specialized training to address these issues

   [ ] Fear of becoming emotionally involved with the patient

   [ ] Few resources available for the subsequent guidance of the patient

   Other____

8. What are the reasons for patients not mentioning their past history of traumatic events or adverse childhood experiences during the consultation?

[ ] Shame

[ ] Refusal

[ ] Fear of not being understood

[ ] Lack of consultation time

[ ] Not important to the consultation

[ ] Not wanting to bother the doctor with these matters

[ ] Prefer to speak with another healthcare professional

[ ] Not wanting to suffer when remembering the events

[ ] Other

Supplementary Material S2

Exploratory factor analysis for the adapted Self-confidence Scale

In our study, we used an adaptation of the Self-confidence Scale. The exploratory factor analysis was used to determine the dimensions for the 11 items selected from the original scale. The Measure of Sampling Adequacy (MSA) of KMO (Kaiser-Meyer-Olkin) produces a statistic whose interpretation is made according to the following Table 1, which indicates the quality of data according to the value obtained.

Table S1. Measure of KMO MSA

	<0.5	0.5-0.6	0.6-0.7	0.7-0.8	0.8-0.9	0.9-1.0
KMO MSA	Unacceptable	Bad	Fair	Average	Good	Very Good

In this case, MSA=0.879 shows the possibility of a factor analysis of the data with good quality. The Bartlett test of sphericity places the null hypothesis that the variables are not correlated, and if this happens, this technique cannot be applied. The results ($\chi^2_{(55)}$=763.6; p<0.001) permit the rejection of the hypothesis that no correlation exists between variables, and therefore we can proceed with the analysis. After checking the possibility of being able to perform factor analysis adequately, the extraction of factors from the 11 variables was made. The communalities extracted, which represent the percentage of common variance of variables in the extracted factors, or saturation, are above the minimum usually required of 40% for all variables, being higher than 52%, values that can explain at least 27% of variance (the variance explained is equal to the square of communality). To determine the number of components to be included in the analysis when using the Kaiser criterion (excludes those components whose Eigenvalues are less than 1 and requires a minimum of 60% for the cumulative extracted variance), three factors should be deducted, which accordingly explain the 69.1% of the total variation in the 11 original variables. The exploratory factor analysis is presented in Table 2.

Table S2. Exploratory factor analysis

	h^2	Factor 1	Factor 2	Factor 3
1. Avoid interrupting the patient	.839	.219	.096	**.884**
2. Avoid making the patient feel rushed.	.758	.242	.308	**.777**
3. Shift the agenda from the patient's to your own at the appropriate time.	.754	.182	**.804**	.274
4. Maintain the flow of the interview.	.765	.270	**.783**	.283
5. Provide an adequate structure for the consultation.	.799	.341	**.826**	.011
6. Identify the patient's non-verbal communication.	.523	**.639**	.203	.271
7. Build a good clinical relationship with the patient.	.639	**.653**	.281	.364
8. Assertively deal with emotions.	.622	**.650**	.437	.095
9. Identify unexpressed feelings for the patient.	.758	**.853**	.146	.094
10. Recognize your own feelings towards the patient (negative or positive).	.544	**.706**	.168	.130
11. Deal with an anxious or depressed patient.	.597	**.646**	.309	.291
Variance explained %		29,4	22.7	17.0
Total variance explained %		69.1		

Rotade component matrix. Extraction Method: Principal Component Analysis. Rotation Method: Varimax with Kaiser Normalization. KMO = 0.879. Factor loadings greater than 0.4 are in bold.

The saturations of the variables in each factor are always above the required minimum of 40%. The following associations between the factors and the variables were presented:

- **Factor 1 - Relationship building,** related to the variables:

6. Identify the patient's non-verbal communication.

7. Build a good clinical relationship with the patient.

8. Assertively deal with emotions.

9. Identify unexpressed feelings for the patient.

10. Recognize your own feelings towards the patient (negative or positive).

11. Deal with an anxious or depressed patient.

- **Factor 2 - Directive facilitation,** related to the variables:

3. Shift the agenda from the patient's to your own at the appropriate time.

4. Maintain the flow of the interview.

5. Provide an adequate structure for the consultation.

- **Factor 3 - Nondirective facilitation,** related to the variables:

1. Avoid interrupting the patient.

2. Avoid making the patient feel rushed.

Supplementary Material S3

Portuguese version of the adapted Self-Confidence Scale

Para abordar acontecimentos traumáticos ou experiências adversas na infância dos seus pacientes, qual o seu grau de confiança em:

1. Evitar interromper o doente?

Nenhuma confiança ○○○○○○○ *Toda a confiança*

1 2 3 4 5 6 7

2. Evitar que o doente sinta que está a ser apressado?

Nenhuma confiança ○○○○○○○ *Toda a confiança*

1 2 3 4 5 6 7

3. Conseguir passar da agenda do doente para a sua própria agenda no momento certo?

Nenhuma confiança ○○○○○○○ *Toda a confiança*

1 2 3 4 5 6 7

4. Conseguir manter o curso da entrevista?

Nenhuma confiança ○○○○○○○ *Toda a confiança*

1 2 3 4 5 6 7

5. Fornecer uma estrutura adequada à consulta?

Nenhuma confiança ○○○○○○○ *Toda a confiança*

1 2 3 4 5 6 7

6. Identificar a comunicação não verbal do doente?

Nenhuma confiança ○○○○○○○ *Toda a confiança*

1 2 3 4 5 6 7

7. Construir uma boa relação clínica com o doente?

Nenhuma confiança ○○○○○○○ *Toda a confiança*

1 2 3 4 5 6 7

8. Lidar assertivamente com emoções?

Nenhuma confiança ○○○○○○○ *Toda a confiança*

1 2 3 4 5 6 7

9. Identificar sentimentos não expressos pelo doente? (ex: "Parece ter sido uma coisa triste para o Sr.")?

Nenhuma confiança ○○○○○○○ *Toda a confiança*

1 2 3 4 5 6 7

10. Reconhecer os seus próprios sentimentos em relação ao doente (negativos ou positivos)?

Nenhuma confiança ○○○○○○○ *Toda a confiança*

1 2 3 4 5 6 7

11. Lidar com um doente ansioso ou depressivo?

Nenhuma confiança ○○○○○○○ *Toda a confiança*

1 2 3 4 5 6 7

Supplementary Material S4

Frequencies related to LEC-5

Column 7 indicates the number of participants who endorsed multiple levels of exposure to the same type of TE. Some participants answered one of the four options of interest, as well as the "Doesn't apply"option, in which case the option "Doesn't apply" was eliminated.

Table S3. Frequencies related with LEC-5 items.

	**1**		**2**		**3**		**4**		**5**		**6**		**7**	
	n	**%**	***n***	**%**	***n***	**%**	***n***	**%**	***n***	**%**	***n***	**%**	***n***	**%**
1. Natural disaster.	13	9.1%	14	9.8%	21	14.7%	1	.7%	6	4.2%	81	56.6%	7	4.9%
2. Fire or explosion.	13	9.1%	23	16.1%	23	16.1%	8	5.6%	4	2.8%	61	42.7%	11	7.7%
3. Transportation accident.	30	21.0%	15	10.5%	27	18.9%	6	4.2%	3	2.1%	47	32.9%	15	10.5%
4. Serious accident at work, home or during a recreational activity.	9	6.3%	6	4.2%	27	18.9%	15	10.5%	2	1.4%	75	52.4%	9	6.3%
5. Exposure to toxic substance.	3	2.1%	2	1.4%	13	9.1%	15	10.5%	9	6.3%	97	67.8%	4	2.8%
6. Physical assault.	14	9.8%	13	9.1%	19	13.3%	21	14.7%	4	2.8%	59	41.3%	13	9.1%
7. Assault with a weapon.	4	2.8%	4	2.8%	19	13.3%	15	10.5%	6	4.2%	90	62.9%	5	3.5%
8. Sexual assault.	3	2.1%	4	2.8%	17	11.9%	20	14.0%	7	4.9%	86	60.1%	6	4.2%
9. Other unwanted or uncomfortable sexual experience.	13	9.1%	3	2.1%	10	7.0%	13	9.1%	7	4.9%	89	62.2%	8	5.6%
10. Combat or exposure to a war-zone.	2	1.4%	4	2.8%	19	13.3%	7	4.9%	4	2.8%	104	72.7%	3	2.1%
11. Captivity.	1	.7%	1	.7%	12	8.4%	2	1.4%	7	4.9%	117	81.8%	3	2.1%
12. Life-threatening illness or injury.	7	4.9%	10	7.0%	13	9.1%	17	11.9%	3	2.1%	72	50.3%	21	14.7%
13. Severe human suffering.	18	12.6%	17	11.9%	6	4.2%	23	16.1%	4	2.8%	55	38.5%	20	14.0%
14. Sudden violent death.	3	2.1%	5	3.5%	17	11.9%	19	13.3%	5	3.5%	84	58.7%	10	7.0%
15. Sudden accidental death.	9	6.3%	9	6.3%	25	17.5%	20	14.0%	5	3.5%	65	45.5%	10	7.0%
16. Serious injury, harm or death you caused to someone else.			2	1.4%	9	6.3%	10	7.0%	8	5.6%	111	77.6%	3	2.1%
17. Any other very stressful event or experience.	23	16.1%	3	2.1%	3	2.1%	4	2.8%	9	6.3%	89	62.2%	12	8.4%

Distribution of the multiple answers for each trauma type of LEC-5

Numbers 1, 2, 3 and 4 correspond to the first four levels of exposure of each trauma type of LEC-5 (1-happened to me, 2- witnessed it, 3- learned about it, 4- part of my job). For example, if the multiple answer is 1 and 2, the code used was 12.

Table S4. Frequencies of multiple answers to the items of LEC-5.

	Multiple answers	N	%
1. Natural disaster.	23	3	2.1
	34	2	1.4
	123	1	.7
	1234	1	.7
2. Fire or explosion.	14	1	.7
	23	5	3.5
	24	2	1.4
	123	2	1.4
	1234	1	.7
3. Transportation accident.	12	1	.7
	13	2	1.4
	14	1	.7
	23	2	1.4
	24	1	.7
	34	2	1.4
	123	1	.7
	124	1	.7
	234	2	1.4
	1234	2	1.4
4. Serious accident at work, at home or during a recreational activity.	23	3	2.1
	34	3	2.1
	123	1	.7
	124	1	.7
	234	1	.7

5. Exposure to toxic substance.	24	1	.7
	34	3	2.1
6. Physical assault.	12	1	.7
	14	1	.7
	23	2	1.4
	24	2	1.4
	34	2	1.4
	123	3	2.1
	1234	2	1.4
7. Assault with a weapon.	24	1	.7
	34	4	2.8
8. Sexual assault.	13	1	.7
	34	4	2.8
	134	1	.7
9. Another uncomfortable or unwanted sexual experience.	34	6	4.2
	123	1	.7
	134	1	.7
10. Combat or exposure to a war zone.	34	1	.7
	124	1	.7
	235	1	.7
11. Forced captivity.	25	1	.7
	34	2	1.4
12. Life-threatening illness or injury.	12	1	.7
	14	1	.7
	23	3	2.1
	24	5	3.5
	34	8	5.6
	234	2	1.4
	1234	1	.7
13. Severe human suffering.	12	1	.7
	14	3	2.1
	23	2	1.4
	24	1	.7
	34	3	2.1

	123	1	.7
	134	1	.7
	234	3	2.1
	1234	4	2.8
	12345	1	.7
14. Sudden, violent death.	24	2	1.4
	34	6	4.2
	234	1	.7
	1234	1	.7
15. Sudden, accidental death.	23	2	1.4
	24	1	.7
	34	4	2.8
	234	2	1.4
	1234	1	.7
16. Serious injury, harm or death caused to someone else.	34	3	2.1
17. Any other stressful event or experience.	12	2	1.4
	14	2	1.4
	15	1	.7
	34	1	.7
	123	2	1.4
	234	1	.7
	1234	3	2.1

Supplementary Material S5

Survey used in our study

INFORMAÇÃO AOS PARTICIPANTES

"Como abordar o historial de acontecimentos traumáticos dos pacientes em Medicina Geral e Familiar: a influência das experiências adversas dos médicos."

Este questionário está integrado num Projeto de Investigação que foi desenvolvido no âmbito do Mestrado em Comunicação Clínica da Faculdade de Medicina da Universidade do Porto. Tem como objetivo principal avaliar a perceção dos Médicos Internos e Especialistas de Medicina Geral e Familiar com prática assistencial em Portugal acerca das suas competências de comunicação clínica na abordagem do historial de acontecimentos traumáticos dos seus pacientes. Pretende, ainda, estabelecer associações entre a forma como estes profissionais de saúde comunicam e a sua história pessoal de experiências adversas.

O preenchimento do questionário requer cerca de 15 minutos, podendo ser interrompido a qualquer momento. A participação nesta investigação é voluntária e não tem qualquer despesa ou incómodo associados. É concedido tempo para reflexão sobre o pedido de participação no estudo.

Nenhuma das questões permite a identificação dos participantes, estando assegurada a privacidade e o anonimato de todos os dados fornecidos. O preenchimento e submissão deste questionário consente a participação no estudo.

Este projeto está aprovado pela Comissão de Ética para a Saúde do Centro Hospitalar Universitário de São João/Faculdade de Medicina da Universidade do Porto.

Grata pela sua participação!

Mariana Sá

Unidade de Psicologia Médica – Departamento de Neurociências e Saúde Mental

Faculdade de Medicina da Universidade do Porto

Médica Interna de Medicina Geral e Familiar

Endereço eletrónico: mariana.oliveira.sa.22@gmail.com

CONSENTIMENTO INFORMADO, ESCLARECIDO E LIVRE

Selecione se aceita participar no estudo:

□ Compreendi a explicação que me foi facultada acerca deste estudo. Solicitei todas as informações de que necessitei, sabendo que o esclarecimento é fundamental para uma boa decisão. Fui informado da possibilidade de livremente recusar a participação no estudo, sem qualquer prejuízo consequente. Declaro que aceito participar neste estudo de forma esclarecida e voluntária.

Confirmo que sou Médico Interno ou Especialista em Medicina Geral e Familiar:

□ Sim

PARTE I – CARACTERIZAÇÃO SOCIODEMOGRÁFICA E PROFISSIONAL

1. Género:

   □ Masculino

   □ Feminino

   □ Outro

2. Data de nascimento: (dia/mês/ano)

3. Categoria profissional:

   □ Médico Interno de Medicina Geral e Familiar

   □ Médico Especialista de Medicina Geral e Familiar

4. Anos de experiência clínica:

   □ < 2 anos

   □ 2 - 5 anos

□ 5 - 10 anos

□ > 10 anos

5. Local onde exerce a sua atividade profissional:

□ USF Modelo A

□ USF Modelo B

□ UCSP

□ Privado

6. Área geográfica onde exerce a sua atividade profissional:

□ Norte

□ Centro

□ Lisboa e Vale do Tejo

□ Alentejo

□ Algarve

□ Região Autónoma dos Açores

□ Região Autónoma da Madeira

7. Formação em Comunicação Clínica:

□ Nenhuma

□ Formação pré-graduada

□ Formação pós-graduada

8. Se assinalou a opção "Formação pós-graduada":

□ Duração (em dias, meses, anos):__________

□ Tipo de formação realizada (pós-graduação, mestrado, outra):_______________

□ Não se aplica

PARTE II – CARACTERIZAÇÃO DE EXPERIÊNCIAS ADVERSAS NA INFÂNCIA E JUVENTUDE

As afirmações seguintes dizem respeito a experiências adversas que muitas vezes ocorrem ao longo da infância e da juventude. Classifique as afirmações de acordo com o que viveu nestas fases da sua vida.

Versão portuguesa do *Childhood Trauma Questionnaire – Short Form* (CTQ-SF)

(Bernstein et al., 2003; Dias et al., 2014)

	**Na minha infância e juventude…**	Nunca	Poucas vezes	Às vezes	Muitas vezes	Sempre
1	Eu não tinha comida suficiente.					
2	Sabia que havia alguém para me cuidar e proteger.					
3	As pessoas da minha família chamavam-me nomes (estúpido (a), preguiçoso (a), feio (a), etc.).					
4	Os meus pais não conseguiam cuidar da família porque se embriagavam ou drogavam.					
5	Havia alguém na minha família que me ajudava a sentir especial ou importante.					
6	Tinha que usar roupas sujas.					
7	Senti-me amado (a).					
8	Achava que os meus pais preferiam que eu nunca tivesse nascido.					
9	Na minha família batiam-me tanto que tinha que ir ao hospital ou ao médico.					
10	A minha família parecia quase perfeita.					

11	Na minha família batiam-me tanto que me deixavam pisado ou com nódoas negras no corpo.					
12	Batiam-me com um cinto, um pau, uma corda ou outras coisas que me magoavam.					
13	As pessoas da minha família cuidavam umas das outras.					
14	Pessoas da minha família diziam coisas que me magoaram ou ofenderam.					
15	Acredito que fui fisicamente maltratado.					
16	Tive uma infância ótima.					
17	Batiam-me tanto que um professor, um vizinho ou um médico chegou a dar-se conta disso.					
18	Sentia que na minha família alguém me odiava.					
19	As pessoas da minha família eram unidas.					
20	Tentaram tocar-me ou obrigaram-me a tocar alguém sexualmente.					
21	Ameaçaram magoar-me ou contar mentiras sobre mim se eu não fizesse algo sexual.					
22	Tive a melhor família do mundo.					
23	Tentaram forçar-me a fazer ou a assistir a algo sexual.					
24	Alguém me assediou.					
25	Acredito que fui maltratado (a) emocionalmente.					
26	Havia alguém para me levar ao médico quando eu precisava.					
27	Acredito que fui abusado sexualmente.					
28	A minha família foi uma fonte de força e apoio.					

PARTE III – CARACTERIZAÇÃO DA HISTÓRIA DE ACONTECIMENTOS TRAUMÁTICOS PRÉVIOS

Versão portuguesa do *Life Events Checklist for DSM-V* (LEC-5)

(Weathers et al., 2013; Ferreira, Ribeiro, Santos & Maia, 2016; unpublished)

<u>Parte 1</u>

Na lista abaixo, encontra um conjunto de situações difíceis ou stressantes que às vezes acontecem. Para cada um dos acontecimentos, assinale uma ou mais das opções à direita, indicando se a situação: **a)** lhe aconteceu pessoalmente; **b)** viu a acontecer a outra pessoa; **c)** soube que aconteceu a um familiar ou amigo próximo; **d)** foi exposto à situação por fazer parte do seu trabalho (ex: paramédico, polícia, militar ou outro profissional de emergência); **e)** não tem a certeza se lhe aconteceu e **f)** não se aplica a si. Ao responder, tenha em consideração toda a sua vida (desde a infância até à idade adulta).

	**Acontecimento**	Aconteceu-me	Vi acontecer	Soube que aconteceu	Parte do meu trabalho	Não tenho a certeza	Não se aplica
1	Desastre natural (ex: inundação, furacão, tornado, terramoto).						
2	Fogo/incêndio ou explosão.						
3	Acidente num transporte (ex: acidente de viação, barco, comboio, avião).						
4	Acidente grave no trabalho, em casa ou durante uma actividade recreativa.						
5	Exposição a substância tóxica (ex: químicos perigosos, radiação).						
6	Agressão física (ex: ser atacado, atingido, esbofeteado, pontapeado, espancado).						
7	Agressão com arma (ser baleado, apunhalado, ameaçado com faca, arma, bomba).						

8	Agressão sexual (ex: violação, tentativa de violação, ser obrigado a praticar qualquer tipo de ato sexual forçado ou sob ameaça).						
9	Outra experiência de cariz sexual desconfortável ou indesejada.						
10	Combate ou exposição a zona de guerra (como militar e/ou civil).						
11	Cativeiro (ex: ser raptado, refém, prisioneiro de guerra).						
12	Doença ou ferimento / lesão com risco de vida.						
13	Sofrimento intenso.						
14	Morte repentina, violenta (ex: homicídio, suicídio).						
15	Morte acidental inesperada violenta.						
16	Causar ferimento grave, dor / lesão ou morte a outra pessoa.						
17	Outro acontecimento ou experiência extremamente stressante.						

Parte 2

A. Se assinalou algo no número 17 da Parte 1, descreva de forma breve o acontecimento a que se estava a referir:

____

____

B. Se experienciou/vivenciou mais do que um acontecimento na Parte 1, pense naquele que considera ser o *pior acontecimento*, o qual será para este questionário o acontecimento que mais o perturba. Se experienciou apenas um acontecimento na parte 1, use esse como referência de "o pior". Por favor, responda às seguintes questões sobre esse acontecimento em específico (assinale todas as opções que se apliquem).

1. Resumidamente, descreva a pior experiência (exemplo, o que aconteceu, quem esteve envolvido, etc.):

2. Há quanto tempo aconteceu (em meses ou anos)?

(se não se recordar faça uma estimativa)

3. Como é que vivenciou a situação?

□ Aconteceu-me diretamente

□ Vi acontecer

□ Soube que aconteceu a um familiar ou amigo próximo

□ Fui exposto de forma recorrente ao acontecimento por fazer parte do meu trabalho (ex: paramédico, polícia, militar ou outro professional de emergência)

□ Outro, por favor descreva:

4. Alguém correu risco de vida?

□ Sim, eu

□ Sim, outra pessoa

□ Não

5. Alguém ficou gravemente ferido ou morreu?

□ Sim, eu fiquei gravemente ferido

□ Sim, outra pessoa ficou gravemente ferida ou morreu

□ Não

6. Envolveu violência sexual?

□ Sim

□ Não

7. Se o acontecimento envolveu a morte de um familiar ou amigo próximo, foi devido a algum tipo de violência, acidente ou devido a causas naturais?

   □ Acidente ou violência

   □ Causas naturais

   □ Não aplicável (o acontecimento não envolveu a morte de um familiar ou amigo próximo)

8. Ao todo, quantas vezes já experienciou um acontecimento tão ou quase tão stressante como este?

   □ Apenas uma vez

   □ Mais do que uma vez (por favor, especifique ou estime o número total de vezes que já teve este tipo de acontecimento____________________________________)

PARTE IV – PERCEÇÃO DOS MÉDICOS ESPECIALISTAS E INTERNOS DE MEDICINA GERAL E FAMILIAR ACERCA DAS SUAS COMPETÊNCIAS DE COMUNICAÇÃO CLÍNICA PARA LIDAR COM EXPERIÊNCIAS TRAUMÁTICAS DOS PACIENTES

As perguntas seguintes permitem refletir acerca do quão à vontade se sente, na sua prática clínica, em abordar e explorar, ao longo da consulta, as experiências traumáticas dos seus pacientes. Selecione a resposta que mais se aproxima da sua realidade.

1. Questiona, por rotina, os seus pacientes acerca da sua história pessoal de exposição a acontecimentos traumáticos e experiências adversas na infância?

   □ Sim

   □ Não

2. Questiona os seus pacientes acerca da sua história pessoal de exposição a acontecimentos traumáticos e experiências adversas na infância apenas em determinados contextos específicos?

   □ Sim

   □ Não

3. Se respondeu "Sim" à pergunta anterior, em que contextos o faz?

   □ Perante sintomatologia depressiva e/ou ansiosa

   □ Perante doença crónica

   □ Perante contexto familiar delicado

   □ Perante situações de multimorbilidade

   □ Perante pacientes muito frequentadores

   Outra:____

4. De quem considera que o início da abordagem desta temática deve partir?

   □ Médico

   □ Paciente

   □ Pode partir de um ou de outro, dependendo da situação

As perguntas seguintes dizem respeito à sua opinião sobre a perspetiva dos pacientes relativamente à abordagem de questões relativas à história pessoal deles de acontecimentos traumáticos e experiências adversas na infância.

5. Quão importante acha que é para os seus pacientes abordar a história deles de exposição a acontecimentos traumáticos e experiências adversas na infância?

   □ Nada importante

   □ Pouco importante

□ Indiferente

□ Importante

□ Muito importante

6. Acha que os seus pacientes gostariam que explorasse rotineiramente a história deles de acontecimentos traumáticos e experiências adversas na infância?

   □ Discordo totalmente

   □ Discordo

   □ Não concordo nem discordo

   □ Concordo

   □ Concordo totalmente

Questionar os pacientes acerca da sua história pessoal de acontecimentos traumáticos e experiências adversas na infância pode ser difícil por várias razões.

7. Quais as razões que identifica para os médicos não abordarem esta temática de forma rotineira?

   □ Vergonha

   □ Recusa

   □ Falta de tempo na consulta

   □ Não ser importante para a consulta

   □ Receio de provocar mais dano ou sofrimento no paciente

   □ Receio de quebrar a relação médico-paciente

   □ Dificuldade em dar uma resposta empática e de contenção perante emoções fortes que surjam durante a partilha do paciente

□ Falta de treino em técnicas de comunicação clínica

□ Sentir que não tem formação especializada para abordar esses tópicos

□ Receio de se envolver emocionalmente com o paciente

□ Poucos recursos disponíveis para uma orientação subsequente do paciente

Outra____

8. Quais as razões para os pacientes não trazerem para a consulta estes temas relacionados com os seus eventos traumáticos ou experiências adversas na infância?

□ Vergonha

□ Recusa

□ Receio de não ser compreendido

□ Falta de tempo na consulta

□ Não ser importante para a consulta

□ Não querer incomodar o médico com esses assuntos

□ Preferir falar com outro profissional de saúde

□ Não querer sofrer ao relembrar os acontecimentos

Outra____

Adaptação da escala de avaliação de autoconfiança quanto às competências de comunicação clínica

(Carvalho et al., 2011; Smith et al., 1995)

Para abordar acontecimentos traumáticos ou experiências adversas na infância dos seus pacientes, qual o seu grau de confiança em:

1. Evitar interromper o doente?

Nenhuma confiança ○○○○○○○ *Toda a confiança*

1 2 3 4 5 6 7

2. Evitar que o doente sinta que está a ser apressado?

Nenhuma confiança ○○○○○○○ *Toda a confiança*

1 2 3 4 5 6 7

3. Conseguir passar da agenda do doente para a sua própria agenda no momento certo?

Nenhuma confiança ○○○○○○○ *Toda a confiança*

1 2 3 4 5 6 7

4. Conseguir manter o curso da entrevista?

Nenhuma confiança ○○○○○○○ *Toda a confiança*

1 2 3 4 5 6 7

5. Fornecer uma estrutura adequada à consulta?

Nenhuma confiança ○○○○○○○ *Toda a confiança*

1 2 3 4 5 6 7

6. Identificar a comunicação não verbal do doente?

Nenhuma confiança ○○○○○○○ *Toda a confiança*

1 2 3 4 5 6 7

7. Construir uma boa relação clínica com o doente?

Nenhuma confiança ○○○○○○○ *Toda a confiança*

1 2 3 4 5 6 7

8. Lidar assertivamente com emoções?

Nenhuma confiança ○○○○○○○ *Toda a confiança*

1 2 3 4 5 6 7

9. Identificar sentimentos não expressos pelo doente? (ex: "Parece ter sido uma coisa triste para o Sr.")?

Nenhuma confiança ○○○○○○○ *Toda a confiança*

1 2 3 4 5 6 7

10. Reconhecer os seus próprios sentimentos em relação ao doente (negativos ou positivos)?

Nenhuma confiança ○○○○○○○ *Toda a confiança*

1 2 3 4 5 6 7

11. Lidar com um doente ansioso ou depressivo?

Nenhuma confiança ○○○○○○○ *Toda a confiança*

1 2 3 4 5 6 7

Versão portuguesa da *Jefferson Scale of Physician Empathy*

(Hojat et al., 2001; Aguiar et al. 2009)

Indique o grau de concordância ou discordância com cada uma das seguintes frases, escrevendo o número apropriado no espaço antes de cada afirmação. Utilize a seguinte escala de sete pontos (o número maior na escala indica maior concordância).

1--------2--------3--------4--------5--------6--------7

Discordo totalmente   Concordo totalmente

1.____A minha compreensão dos sentimentos dos meus pacientes e das suas famílias é um fator irrelevante para o tratamento médico ou cirúrgico.

2.____Os meus pacientes sentem-se melhor quando eu compreendo o que eles sentem.

3.____É difícil para mim ver as coisas pela perspetiva dos meus pacientes.

4.____Considero que, na relação médico doente, compreender a linguagem do paciente é tão importante quanto a comunicação verbal.

5.____Tenho um bom sentido de humor que, penso, contribui para um melhor resultado clínico.

6.____Pelo facto das pessoas serem diferentes, é difícil para mim ver as coisas na perspetiva dos meus pacientes.

7.____Tento não prestar atenção às emoções dos meus pacientes nas entrevistas e na colheita de histórias clínicas.

8.____A atenção que dedico às experiências pessoais dos meus pacientes não influencia os resultados de tratamento.

9.____Tento imaginar-me no lugar dos meus pacientes quando estou a cuidar deles.

10.____A minha compreensão dos sentimentos dos meus pacientes dá-lhes, a eles, uma sensação de legitimação que é terapêutica em si mesma.

11.____As enfermidades dos doentes podem ser curadas apenas por tratamento médico ou cirúrgico; consequentemente, os laços afetivos que tenha com os meus pacientes não podem ter uma influência significativa nos resultados médicos ou cirúrgicos.

12.____Considero que questionar os pacientes relativamente ao que se passa na sua vida pessoal é um fator sem importância para a compreensão das suas queixas de ordem física.

13.____Tento compreender o que se passa na mente dos meus pacientes, prestando atenção à comunicação não verbal e linguagem corporal.

14.____Creio que as emoções não têm lugar no tratamento da doença médica.

15.____A empatia é uma competência terapêutica sem a qual o sucesso no tratamento é limitado.

16.____Uma componente importante do relacionamento com os meus pacientes é a minha compreensão do seu estado emocional e o das suas famílias.

17.____Tento pensar como os meus pacientes, para que lhes possa prestar melhores cuidados.

18.____Não me deixo influenciar por ligações fortes de natureza pessoal entre os pacientes e os seus familiares.

19.____Não aprecio arte nem literatura que não seja médica.

20.____Acredito que a empatia é um fator terapêutico importante no tratamento médico.
